# An Update on Post‐Surgical Wound Healing Management and Lower‐Cost Alternatives: A Narrative Review for Healthcare Providers

**DOI:** 10.1002/hsr2.73353

**Published:** 2026-10-07

**Authors:** Joseph D. Quick, Lauren E. Powell, Aneesh Syal, Neej N. Patel, Lana Kamel, Athena Brooks, Syed Ali Haider, Timothy A. Powell, Marie‐Claire Buckley

**Affiliations:** ^1^ Cleveland Clinic Lerner College of Medicine Cleveland Ohio USA; ^2^ Division of Plastic Surgery University of Minnesota Minneapolis Minnesota USA; ^3^ Department of Pediatrics East Carolina University Greenville North Carolina USA

**Keywords:** education, health equity, lower‐cost care, wound, wound care, wound dressing

## Abstract

**Background and Aims:**

Wound care is a critical component of postoperative and chronic disease management, yet rising supply costs, inconsistent insurance coverage, and patient‐level barriers can complicate care in outpatient and home settings. This narrative review summarizes wound‐management principles and evaluates lower‐cost alternatives, emphasizing differences in evidence strength, appropriate wound contact, contraindications, and required clinical oversight.

**Methods:**

A narrative review of English‐language literature available through July 2026 was conducted using PubMed/MEDLINE, Google Scholar, Scopus, Web of Science, and reference‐list review. Evidence from guidelines, systematic reviews, meta‐analyses, randomized trials, and clinical studies was prioritized. Evidence supporting each lower‐cost strategy was categorized qualitatively by study design and directness.

**Results:**

Evidence was strongest for potable tap‐water cleansing in selected wounds, plain petrolatum for selected clean postprocedural wounds, and paper tape for selected closed incisions. Nonmedical absorbent products have a limited role as external secondary protection and should not contact open wound beds. Clinician‐assembled negative‐pressure wound therapy and nonmedical compression require trained selection and monitoring. Raw honey, direct application of essential oils, and unsupervised homemade wound solutions are not recommended.

**Conclusion:**

Certain lower‐cost wound care products may expand access to care for patients facing financial or logistical barriers. However, these alternative products should preserve the same safety threshold as conventional care and should be selected according to wound type, depth, exudate, perfusion, infection status, and treatment goal. Further comparative research is needed to establish the effectiveness, safety, and cost savings of alternative wound care strategies.

## 1 Introduction

Wound care is a cornerstone of postoperative recovery in all surgical and interventional specialties. Optimal wound management is essential not only for preserving form and function but also for ensuring patient satisfaction. However, the increasing economic burden associated with wound care, both for health systems and individual patients, has called into question the feasibility and sustainability of traditional paradigms.

In recent years, the United States has seen a rise in chronic wounds and wound complications, a trend exacerbated by the growing prevalence of diabetes, obesity, and an aging population [1, 2]. A 2018 study estimated that total Medicare spending for chronic wound care ranges from $28.1 to $96.8 billion annually, with surgical wounds accounting for a substantial proportion of this financial burden [1]. Nonhealing wounds can significantly alter the cosmetic or functional result and thus necessitate high‐quality care to ensure satisfactory outcomes [2, 3, 4]. At the same time, many patients face logistical and economic challenges in maintaining proper wound care, especially at home.

Compounding this issue is the often‐confusing landscape of wound care products, ranging from advanced biologic dressings to different types of gauze, many of which are proprietary, expensive, and sometimes complex [5]. There is a need to identify lower‐cost approaches that preserve established standards of safety and effectiveness, particularly for home care in resource‐constrained settings. In this review, lower‐cost and cost‐conscious refer to acquisition price, availability, or reduction of a discrete expense. They do not imply formal cost‐effectiveness, which would require comparison of clinical outcomes, complications, dressing‐change frequency, durability, clinician and caregiver time, and total episode‐of‐care costs. The authors review challenges of home wound care, summarize traditional management, and distinguish evidence‐supported economical approaches from options supported only by indirect evidence or expert opinion.

## 2 Challenges of Home Wound Care

The financial cost of wound care presents a major challenge for patients, particularly in cases where wounds require more specialized management. These costs arise through three main pathways: gaps in insurance coverage, direct costs of supplies, and indirect costs associated with wound healing (i.e., time away from work, travel expenses, home modifications). Insurance coverage for wound care is complex and can be patchy, particularly when the care occurs at home. Currently, Medicare is the largest third‐party payer for patients with chronic wounds [6]. Medicare Part B covers surgical dressings when used on a qualifying wound, which is defined as a wound that is surgically created, treated, or debrided [7]. However, this coverage is limited to specific dressing types, quantities, and frequencies as outlined by the Durable Medical Equipment Medicare Administrative Contractors (DME MACs) [7]. Coverage does not extend to all commercially available products, nor does it typically include ancillary items like skin sealants or barriers, topical antiseptics/antibiotics, or surgical stockings [7]. Patients must also obtain supplies through a Medicare‐enrolled supplier, and reimbursement often requires a physician's order and documentation of wound characteristics, frequency of dressing changes, and debridement status at least every 3 months, making this a highly sophisticated process [7]. If these criteria are not met, patients may be responsible for all associated costs. Private insurance plans and other public programs often mirror these restrictions, though policies vary widely. Many patients undergoing elective surgery may find that their insurer does not cover dressing supplies at all, considering them “custodial” or “comfort” items. In such cases, patients are often responsible for purchasing all wound care products out‐of‐pocket, likely contributing to the increase in predicted out‐of‐pocket expenses [8]. Moreover, requirements for prior authorization and documentation of wound characteristics can delay access to supplies even when coverage is technically available.

The direct out‐of‐pocket cost of wound care supplies can be substantial. Postoperative wound care often involves multiple dressing layers, frequent changes, and the use of specialized products to maintain a clean healing environment. Sterile gauze pads, dressings, bandages, adhesive tapes, and topical antimicrobial agents each carry individual costs that quickly accumulate. While the exact cost of managing a wound varies significantly, estimates show that simple, uncomplicated wounds can cost around $100 weekly, whereas treating surgical site infections (SSIs) can increase that figure to tens of thousands of dollars [9]. Cost constraints may result in inadequate or improper dressing changes, thereby increasing the risk of complications, delaying healing, and ultimately leading to higher downstream healthcare utilization [10].

Taken together, these considerations can affect a patient's ability to adhere to wound care. Table 1 summarizes health‐literacy challenges and communication strategies. Table 2 lists selected prescription‐access programs that may reduce the purchase price of prescribed products; their prices, eligibility, and availability change over time, and inclusion is not an endorsement or evidence of cost‐effectiveness.

**Table 1:** Health Literacy Problems and Potential Solutions Pertaining to Wound Healing.

Health Literacy Problem	Solution
Patients discharged with limited understanding of wound care instructions	Provide clear, step‐by‐step wound care guides using plain language and visual aids
Instructions provided only verbally or in complex written language	Use teach‐back methods and supplement verbal instructions with multimodal diagrams or videos
Low baseline health literacy among older adults and those with limited education	Assess health literacy pre‐discharge using validated tools and provide targeted educational support
Language discordance between provider and patient	Use professional medical interpreters and provide translated wound care materials
Cognitive impairment, stress, or sedation reducing comprehension	Schedule follow‐up calls or home visits to reinforce understanding post‐discharge
Postoperative instructions often not adapted to patients' reading level	Write discharge materials at a 6th‐grade reading level and test for readability
Patients unaware of signs of infection or wound dehiscence	Educate patients using visual symptom charts, mobile applications, and offer hotline access for concerns
Improper dressing changes or product application due to misunderstanding	Demonstrate dressing changes during the hospital stay and provide simplified written or video guides

**Table 2:** Selected Prescription‐Access Resources Relevant to Wound Care.

Program	Website	Current function	Counseling and limitations
Cost Plus Drugs	https://www.costplusdrugs.com/	Online pharmacy using transparent acquisition cost plus a 15% markup and pharmacy labor fee; shipping and taxes vary.	Prescription and account required; not every medication is available; compare the final delivered price with insurance and local pharmacy prices.
GoodRx	https://www.goodrx.com/	Free coupons and price comparison for prescriptions and some prescribed over‐the‐counter products at participating pharmacies.	A coupon is used instead of insurance and does not count toward the deductible; prices and pharmacy participation can change.
SingleCare	https://www.singlecare.com/	Free prescription‐discount coupons and pharmacy price comparison at participating pharmacies.	Not insurance and cannot be combined with insurance for the same purchase; verify the current cash, copay, and coupon prices.
Blink Health/BlinkRx	https://www.blinkhealth.com/	Online prescription‐pricing and fulfillment services; available pickup or delivery pathways depend on the medication and location.	Not insurance; eligibility, fulfillment pathway, and price vary; confirm current terms before counseling a patient.
MedShare	https://www.medshare.org/	Nonprofit distribution of donated medical supplies to eligible health systems and partner clinics.	Generally not a direct‐to‐consumer source; patients should be referred through a participating clinic or local assistance program.

*Note:* Websites and general program functions were verified September 2, 2026. Prices, eligibility, pharmacy participation, and availability can change. These programs address prescription purchase price rather than wound‐dressing cost, and inclusion does not constitute endorsement. Patients should compare the final cash, insurance, and coupon prices at the point of care.

## 3 Methods

This narrative review was conducted to summarize evidence on postoperative and chronic wound management and to evaluate practical, lower‐cost alternatives to conventional products. Searches were conducted iteratively in PubMed/MEDLINE, Google Scholar, Scopus, and Web of Science, supplemented by reference‐list review, for English‐language literature available through July 2026. Search concepts combined wound population terms (“wound healing,” “postoperative wound care,” “surgical wound,” and “chronic wound”) with intervention terms (“wound dressing,” “wound cleansing,” “debridement,” “negative pressure wound therapy,” and “scar management”) and access terms (“cost,” “affordability,” “health literacy,” “home wound care,” and “alternative wound care”). Boolean operators and database‐specific syntax were adapted during the iterative search.

Priority was given to clinical practice guidelines, systematic reviews, meta‐analyses, randomized controlled trials, and comparative clinical studies. Seminal studies were retained when needed for foundational physiology or techniques. Eligible sources addressed human wound assessment, cleansing, dressings, topical therapy, negative‐pressure therapy, compression, scar management, access, safety, or cost. Animal and in vitro studies, conference abstracts, non‐English publications, and sources lacking sufficient methodological detail were excluded from clinical recommendations; nonclinical sources were used only for mechanistic context. Candidate sources were selected for relevance to clinical decision‐making; no formal duplicate screening or adjudication process was used.

Evidence supporting lower‐cost strategies was synthesized using four descriptive categories: higher‐level evidence (guidelines or systematic reviews), comparative clinical evidence, limited clinical evidence (observational studies, case series, or technical reports), and indirect evidence or expert opinion. These categories describe study design and directness but do not constitute a formal GRADE assessment. Table 3 applies these categories and identifies indication, permitted wound contact, contraindications, and required supervision. Recommendations were narrowed when the cited source did not directly support the population, wound type, product, or outcome described [31].

**Table 3:** Clinical Decision Matrix for Lower‐Cost Wound‐Care Options.

Clinical function and option	Evidence strength and directness	Indication and wound contact	Contraindications and required supervision	Cost interpretation
External secondary absorption ‐ fragrance‐free menstrual or incontinence pad; selected clean diaper material, animal‐training pad, or microfiber cloth	Limited clinical evidence for feminine hygiene pads; indirect functional rationale only for other household materials [11]	Menstrual or incontinence pads may be used outside a sealed medical‐grade primary dressing or against intact intertriginous skin. Other listed materials have only a theoretical external role when the primary dressing fully isolates them from the wound and a clinician approves use.	Nonsterile; never place on an open wound bed. Avoid fragrance, lotion, exposed absorbent gel, loose fibers, or exposed adhesive. Diaper material, animal‐training pads, and microfiber cloths are not clinically validated wound‐care products.	Usually inexpensive, but theoretical external use does not establish clinical equivalence or total‐episode value.
Small‐area external cushioning ‐ cosmetic cotton round	Indirect evidence or expert opinion; no comparative wound‐care trials	External padding over an intact primary dressing for a small, low‐drainage site when medical secondary padding is unavailable.	Do not use as a primary dressing or allow fibers to contact an open wound. Discontinue for irritation or retained fibers; clinician approval is advised.	Low unit price does not establish clinical equivalence.
Cleansing ‐ potable tap water	Higher‐level evidence ‐ systematic review; certainty varies by wound type [12]	Selected low‐risk wounds when local water quality is suitable and the treating clinician's protocol permits.	Use sterile solution when required by wound depth, exposed structures, host risk, contamination, or protocol. Home‐mixed saline is not preferred because concentration and contamination are difficult to control.	Minimal acquisition cost; downstream outcomes depend on wound and patient selection.
Moisture barrier ‐ plain petrolatum	Comparative clinical evidence in selected clean postprocedural wounds [13]	Thin layer for selected clean, closed or superficial postprocedural wounds when approved by the procedural clinician.	Not a treatment for infection, dehiscence, deep wounds, or wounds requiring a prescribed antimicrobial; stop for sensitivity.	Often less expensive than topical antibiotics, but formal total‐episode cost‐effectiveness was not assessed.
Clinician‐directed antiseptic ‐ commercial dilute acetic acid or stabilized HOCl	Comparative or limited clinical evidence; wound models and indications differ [14, 15]	Selected colonized or infected chronic wounds for dilute acetic acid, or selected acute wounds for a stabilized HOCl formulation, only under a clinician‐directed protocol.	Do not extrapolate across wound types or use homemade formulations. Concentration, formulation, contact time, duration, and reassessment must be clinician‐directed.	No general acquisition‐cost or cost‐effectiveness advantage was established.
Medical‐grade honey dressing	Higher‐level evidence ‐ systematic reviews with heterogeneous indications and comparators [16, 17]	Only a sterile, standardized medical‐grade product for a clinician‐selected wound with an appropriate primary dressing plan.	Raw, farmers‐market, or food‐grade honey must not be applied to wounds because it is nonsterile and unstandardized and may contain microbial spores; consider allergy and exudate management.	Medical‐grade products may not cost less than standard dressings; no general cost advantage is assumed.
Standardized aloe vera product	Higher‐level evidence ‐ heterogeneous systematic review, strongest for selected superficial burns [18]	Only for a superficial, clinician‐assessed indication using a standardized commercial formulation.	Do not use fresh plant material or homemade preparations on surgical, deep, infected, or complex wounds; do not delay burn evaluation.	Purchase price may be low, but comparative effectiveness and total cost remain uncertain.
Essential oils	Indirect or preclinical evidence; documented contact‐allergy risk [19, 20]	No routine direct‐wound role outside a research protocol or regulated formulation prescribed for a specific indication.	Variable composition, irritant or allergic contact dermatitis, and possible cytotoxicity; never use undiluted oils on an open wound.	Low retail price does not justify use when safety and effectiveness are unestablished.
Clinician‐assembled gauze‐based NPWT	Limited clinical evidence ‐ small observational series and clinical review [21, 22, 23]	Only by trained clinicians in a monitored institutional setting when commercial NPWT is unavailable and the wound is appropriate.	Never an unsupervised home substitute. Avoid or protect exposed vessels, organs, nerves, anastomoses, unexplored fistulae, necrotic eschar, untreated osteomyelitis, active bleeding, and other contraindications. Document pressure and dressing counts.	Material price may be lower, but staffing, monitoring, complications, and total‐episode cost were not established.
Household plastic wrap	Limited clinical evidence ‐ microbiologic study of temporary acute‐burn coverage, not postoperative treatment [24]	At most, temporary acute‐burn protection under an established protocol or an external shower barrier over a fully sealed primary dressing.	Not equivalent to medical film. Do not use for routine direct contact with postoperative or chronic wounds, prolonged occlusion, infected wounds, or unsupervised home treatment.	Very low purchase price; clinical equivalence and total cost are unproven.
External foam guard for a reconstructed nipple	Limited clinical evidence ‐ single technical report [25]	External mechanical protection according to the operating surgeon's instructions, with the primary dressing intact.	Not a primary dressing and not for direct contact with an open or infected wound; monitor for pressure, shear, or adhesive injury.	Low material cost in the published technique; evidence applies only to this narrow indication.
Paper tape for a closed incision	Comparative clinical evidence ‐ randomized study and review [26, 27]	Tension offloading over a fully epithelialized, clean linear incision in a clinician‐selected patient.	Do not apply to an open, infected, draining, or fragile wound. Avoid known adhesive allergy and discontinue for dermatitis or blistering.	Generally lower acquisition cost than silicone, but adherence and skin tolerance affect value.
Over‐the‐counter elastic wrap or compression garment	Indirect evidence plus higher‐level safety consensus [28, 29, 30]	Only after clinician assessment of indication, perfusion, sensation, fit, and target pressure, with follow‐up monitoring.	Not an automatic substitute for a measured medical garment. Avoid unsupervised use when arterial perfusion is impaired or uncertain, sensation is reduced, fit is uneven, or cardiopulmonary status is unstable.	Lower purchase price does not establish equivalent pressure delivery, durability, outcomes, or cost‐effectiveness.

*Note:* Evidence strength is described using four qualitative categories: higher‐level evidence (guidelines or systematic reviews), comparative clinical evidence, limited clinical evidence (observational studies, case series, or technical reports), and indirect evidence or expert opinion. These categories describe study design and directness and are not formal GRADE ratings. Direct wound contact means contact with an open wound bed. Nonsterile household products should not contact an open wound bed. Cost statements refer only to acquisition price; formal cost‐effectiveness and total episode‐of‐care costs were not established.

Abbreviations: HOCl, hypochlorous acid; NPWT, negative‐pressure wound therapy.

The review was not prospectively registered and was not designed as a systematic review. Exact database‐specific search logs, formal record counts, risk‐of‐bias scoring, and meta‐analysis were not performed. These limitations restrict reproducibility and preclude claims of exhaustive evidence capture or comparative effectiveness.

### 3.1 Basics of Traditional Wound Healing Management by Wound Healing Stage

Wound healing proceeds through overlapping stages of hemostasis, inflammation, proliferation, and remodeling that restore tissue integrity after injury [32]. Effective wound healing begins with principles that apply across wound types and settings. Although advanced therapies and surgery are necessary for some wounds, many can be managed through consistent attention to basic wound care. The T.I.M.E. framework addresses four components of wound‐bed preparation: Tissue management/debridement, Inflammation and Infection control, Moisture balance, and Edge advancement [5].

The four‐stage framework is used here to explain physiology, not as the primary determinant of product selection. In practice, clinicians should first distinguish a clean, closed incision from an open acute wound, chronic ulcer, burn, pressure injury, infected wound, donor site, or complex reconstructive wound. Dressing and adjunct selection should then be based on depth, tissue viability, exudate, perfusion, contamination or infection, exposed structures, surrounding skin, patient risk, and the treatment goal. Recommendations should not be transferred across wound types solely because wounds occupy a similar biological phase.

#### 3.1.1 Stage 1: Hemostasis

In surgical wounds, hemostasis is often mechanically assisted through ligation, cautery, or topical hemostatic agents [33]. Anticoagulation, coagulopathy, or excessive tissue trauma may disrupt this phase and increase hematoma or seroma risk [34]. Dressings applied after hemostasis should contain drainage, protect the wound, and permit recognition of recurrent bleeding. In chronic or nonhealing wounds stalled by devitalized tissue or biofilm, clinician‐directed surgical or enzymatic debridement may remove nonviable tissue, reduce bioburden, and help reinitiate healing [35]. Because chronic wounds may not develop normal collagen architecture, they can remain vulnerable even after apparent closure. Dressings can also manage exudate, protect against mechanical trauma, and support temperature, hydration, and pH.

Sterile gauze is commonly used immediately after clean, closed surgery to cover sutures, absorb limited blood or drainage, and protect the surface [36, 37]. These passive dressings are inexpensive, available, and adaptable, but they do not actively regulate moisture or provide an antimicrobial effect. Gauze may require frequent changes when drainage persists and can adhere to an open granulating wound, potentially disrupting tissue during removal [36, 38, 39]. Its role therefore differs between a closed postoperative incision and an open wound.

For open wounds with brisk exudate or modest bleeding, alginate dressings are typically a superior alternative. Alginate dressings, made from calcium alginate fibers derived from seaweed, are highly absorbent and convert to a gel upon contact with exudate [36]. In addition to absorbing fluid, alginates have mild hemostatic properties through their calcium content, which supports platelet activation and clot stability [36]. Their mild hemostatic properties are beneficial following debridement or skin grafting [40]. In fact, alginates are the most common donor‐site wound dressings for split‐thickness skin grafts [41]. These dressings are also particularly useful for deep pressure ulcers, pyoderma gangrenosum, and exudative ulcers or other areas where absorbency is a primary concern [40].

Other advanced dressings applied during the hemostatic phase include film dressings. Film dressings, consisting of thin polyurethane sheets, are commonly used to protect clean surgical incisions, donor sites for split‐thickness skin grafts, and superficial lacerations [36, 40, 42]. They are semi‐permeable, allowing gas exchange while blocking bacteria and external contaminants [36]. These transparent films do not absorb fluid but instead act as a protective barrier over clean, dry incisions, maintaining sterility while allowing wound inspection [39]. Their use is thus appropriate once active bleeding has ceased but before exudate output has become significant. The transparency of films allows for continuous monitoring of the wound site, making them ideal for wounds in visible areas where minimal disruption to the dressing is desirable [42]. While operative methods ensure primary control of bleeding, the judicious use of simple gauze, alginates, and/or films plays a key role in wound cleanliness and progression to the next phase of healing.

#### 3.1.2 Stage 2: Inflammation

During inflammation, care focuses on minimizing tissue trauma, controlling bacterial burden, and performing debridement when indicated. Cleansing, which may also occur during hemostasis, removes surface debris, exudate, dried blood, and potential pathogens without injuring viable tissue or delaying re‐epithelialization. For selected clean, closed surgical wounds and uncomplicated open acute wounds, gentle irrigation with normal saline or potable water is appropriate, although cleansing does not necessarily accelerate healing [12, 43]. Deep, contaminated, infected, or complex wounds and wounds with exposed critical structures require condition‐specific, clinician‐directed cleansing.

The routine use of antiseptic solutions remains controversial. Cytotoxicity depends on the agent, formulation, concentration, exposure time, wound type, and clinical indication. Chlorhexidine and hydrogen peroxide can damage viable cells in experimental models, but this does not justify a blanket prohibition across all formulations or indications [43]. When an antiseptic is clinically indicated for a contaminated or infected wound, the least cytotoxic effective formulation and duration should be selected, with reassessment as the wound progresses.

Topical antimicrobials may be considered for wounds with a defined risk of contamination or infection, but their benefit and cytotoxicity vary by product and indication. Mupirocin, iodine‐based products, and selected silver formulations have roles in specific clinical contexts [44, 45]. Silver sulfadiazine remains used in some burn settings, but systematic reviews of partial‐thickness burns report slower healing and greater dressing burden than several modern alternatives; it should not be presented as a universally beneficial standard [46]. Prolonged or routine antimicrobial use should be avoided when there is no clear indication, and suspected infection should prompt clinical assessment rather than unsupervised product substitution.

Debridement should be limited to wounds with devitalized tissue and selected according to mechanism and wound characteristics. Collagenase provides enzymatic debridement, whereas hydrophilic pastes, polymeric membrane dressings, and hypertonic saline dressings serve distinct moisture or exudate‐management functions; they should not be assumed to share collagenase's mechanism or cellular effects [47]. Clinician‐directed enzymatic debridement should be stopped when it is no longer needed, including when granulation tissue is established. These measures address the tissue and inflammation/infection components of T.I.M.E. and are not routine treatments for clean, closed incisions.

#### 3.1.3 Stage 3: Proliferation

Successful management of the proliferative phase, especially in open wounds, involves supporting moisture balance, protecting new tissue, and accelerating granulation and epithelial migration. Dressing selection is particularly critical during this stage, as the wound environment must remain adequately moist without excessive exudate accumulation or trauma during dressing changes.

As the wound transitions from the inflammatory phase to active tissue regeneration, hydrocolloid dressings can be used to support this shift. Hydrocolloids are composed of carboxymethylcellulose, gelatin, and pectin particles within an adhesive matrix [42]. They create a gel as they absorb wound fluid and maintain a moist, occlusive environment [42]. These dressings provide mild cushioning, stimulate autolytic debridement, and are best suited for superficial wounds with low‐to‐moderate exudate, including abrasions and postoperative, pressure, burn, and traumatic wounds [40, 42]. Due to their occlusive nature, hydrocolloids are not appropriate for infected or heavily exudative wounds, but when properly selected, they offer a low‐maintenance dressing option that supports the biological goals of progression into granulation and re‐epithelialization while reducing dressing change frequency and patient discomfort [40, 42, 48].

Foam dressings absorb moderate‐to‐heavy exudate while maintaining a moist, thermally insulated environment [36, 37]. Polyurethane or silicone foams are widely used for open surgical wounds and can provide cushioning in pressure‐prone areas or over bony prominences. Their absorptive capacity may reduce maceration and dressing‐change frequency. Their role nevertheless depends on drainage, depth, surrounding skin, and whether the wound is an open defect or a closed incision; they are not uniformly preferable across wounds in the proliferative phase.

Hydrogels donate moisture and may support autolytic debridement or pain relief in selected dry or minimally exudative wounds, including some superficial burns and ulcers [36, 37]. They are inappropriate for heavily exudative wounds and should not be used as stand‐alone treatment for infected wounds. Evidence for healing is indication‐specific: small trials suggest possible benefit for diabetic foot ulcers compared with basic contact dressings, whereas evidence for venous leg ulcers remains inconclusive [49, 50].

Beyond dressings, negative pressure wound therapy (NPWT), or vacuum‐assisted wound closure (VAC), plays a prominent role during the proliferative phase. NPWT is a key tool used in managing large or complex open surgical wounds. By applying sub‐atmospheric pressure through a sealed foam interface, NPWT removes exudate, reduces peri‐wound edema, promotes angiogenesis, and accelerates granulation tissue formation [21]. NPWT is frequently used to bolster skin grafts, prepare wound beds for flap coverage, and manage dehisced incisions or pressure ulcers [21].

Although less widely used, hyperbaric oxygen therapy (HBOT) plays a specialized role in the treatment of ischemic wounds, compromised flaps and grafts, and chronic radiation injuries [51]. HBOT works by enhancing oxygen delivery to hypoxic tissues and has been shown to improve healing in select patients, such as those undergoing extremity replantation and free tissue transfer [51]. However, HBOT is resource‐intensive and not widely accessible to many. Furthermore, additional research is needed to clarify which patient populations benefit from HBOT and how these patients can be identified. Overall, clinical decisions during this stage, such as dressing selection and adjunctive therapies, should be guided by the wound's appearance, location, and drainage pattern, with the overarching goal of transitioning the wound into the remodeling phase with minimal complications.

#### 3.1.4 Stage 4: Remodeling

Remodeling is the final and longest phase of wound healing, beginning a couple of weeks after injury and continuing for months to years [32]. The quality of the remodeling phase is highly significant and determines scar appearance, pliability, and long‐term durability [52]. Hypertrophic scars, keloids, and atrophic scars are all the result of abnormal remodeling dynamics [52]. Surgical technique, mechanical offloading, silicone sheeting, and pressure therapy may be employed postoperatively to modulate this phase and optimize outcomes [52].

Mechanical tension contributes to pathological scarring, particularly in high‐stress areas [53]. During the first postoperative week, the wound depends largely on the fibrin clot and sutures. Tensile strength then increases most rapidly through approximately 6 weeks and plateaus near 3 months [54]. Notably, even fully healed skin does not return to baseline, and seminal studies have demonstrated that maximum tensile strength reaches only about 80% of that of uninjured tissue [54, 55]. Diabetes, malnutrition, and chronic corticosteroid exposure may further impair remodeling and increase vulnerability to later dehiscence or ulceration [56]. Layered, tension‐reducing closure, appropriate suture selection, and incision placement along relaxed skin‐tension lines can limit mechanical stress. Tape reinforcement or another clinician‐selected tension‐offloading method may further support selected clean, closed incisions during early remodeling [57, 58, 59].

Silicone sheeting and sprays are additional interventions used in the remodeling phase. They function by creating a stratum‐corneum‐like semi‐occlusive barrier over the healing wound, which modulates hydration of keratinocytes, reduces capillary activity, and decreases fibroblast activity and collagen deposition [60, 61]. Some clinical trials have demonstrated that silicone sheeting can significantly reduce the incidence and/or severity of hypertrophic scarring and keloid formation, particularly when applied during the early remodeling phase to high‐risk wounds [62]. Limited studies with silicone gel have shown it to produce significant reductions in texture, color, and height of scars as well [60, 62, 63]. However, the current body of evidence is still limited [62].

Another mainstay of scar modulation is pressure therapy, which has been used in the management of burns and large traumatic injuries [64]. The mechanism of pressure therapy involves creating a hypoxic environment by exerting perpendicular and circumferential forces on the skin, which inhibits fibroblast growth and differentiation [64]. Pressure therapy is most effective when initiated early, within 2 months after injury [64]. While somewhat intensive, it remains a critical adjunct in the prevention of contractures and thickened scars. As a whole, early recognition of abnormal scarring patterns and proactive interventions can dramatically influence remodeling outcomes.

### 3.2 Ethical and Equity Considerations

Affordability should be incorporated into shared decision‐making without creating a lower safety standard for patients with fewer resources. Clinicians should explain whether an option is evidence‐supported, supported only indirectly, or not recommended; offer the conventional standard when it is clinically necessary; and document the rationale, instructions, monitoring plan, and thresholds for escalation. A lower purchase price should not justify an option that increases infection, tissue injury, delayed healing, caregiver burden, or downstream utilization. Cost discussions should be conducted privately and without assumptions about a patient's means. Plain‐language counseling, professional interpretation, teach‐back, and demonstration can reduce stigma and prevent unsafe improvisation. Health systems should first use coverage navigation, enrolled suppliers, social work, charitable programs, and standardized low‐cost formularies before asking patients to repurpose household materials.

### 3.3 Lower‐Cost Wound‐Care Strategies by Clinical Function and Wound Characteristics

Lower purchase price can improve access, but it cannot compensate for inappropriate product selection. Any alternative should match the clinical function of the conventional product, preserve a safe wound‐contact layer, and be used only when its evidence and risks are understood for the wound type and patient. Table 3 applies standardized evidence categories and summarizes indication, wound contact, contraindications, supervision, and cost interpretation of the various dressings discussed.

#### 3.3.1 External Absorption and Secondary Protection

Fragrance‐free menstrual or incontinence pads may be considered only as external secondary absorbers over a sealed medical‐grade primary dressing or against intact intertriginous skin. A limited clinical report describes feminine hygiene pads in intertriginous wound care, but this does not establish equivalence to sterile nonadherent or foam dressings [11]. These products are not sterile, may contain fragrances or adhesives, and should not contact an open wound bed. Cosmetic cotton rounds may provide temporary external cushioning over a small, intact primary dressing when medical secondary padding is unavailable. They should not be used as a primary dressing because fibers can adhere to tissue and the product is not sterile. Any nonmedical external absorber should be fragrance‐free, kept outside the wound‐contact layer, changed when wet, and discontinued if irritation develops. Animal‐training pads, clean diaper materials, and microfiber cloths do not have direct clinical evidence but may serve a role similar to that of feminine hygiene pads and cotton rounds.

#### 3.3.2 Cleansing and Topical Products for Selected Low‐Risk Wounds

For selected low‐risk wounds, potable tap water is the best‐supported low‐cost alternative to commercial saline. A 2022 Cochrane review found no clear evidence that cleansing with tap water increases infection compared with saline, although certainty varies by wound type and study [12]. Water quality, wound depth, exposed structures, immune status, and local protocol remain important. Home‐prepared saline is not routinely preferable to potable tap water or commercially sterile saline because salt concentration and contamination are difficult to control. If no safe water or sterile product is available, preparation should follow a standardized public‐health or institutional protocol rather than an improvised recipe.

For selected clean, closed, or superficial postprocedural wounds, plain petrolatum can maintain moisture without routine antibiotic exposure. Comparative clinical evidence suggests healing outcomes similar to those with combination antibiotic ointment in selected settings, while topical antibiotics may cause allergic contact dermatitis [13]. Petrolatum is not a treatment for infection, dehiscence, deep wounds, or wounds for which a clinician has prescribed an antimicrobial, and patients should follow procedure‐specific instructions.

Suspected bacterial or fungal infection should prompt clinical assessment rather than substitution with household or over‐the‐counter products. Antifungal foot powders are intended for labeled use on intact skin and should never be poured into an open wound. Dilute acetic acid has antibiofilm evidence in selected chronic wounds, whereas stabilized hypochlorous acid has limited clinical evidence in experimentally created acute wounds; neither should be generalized across wound types [14, 15]. Concentration, formulation, exposure, and duration should be clinician‐directed. Homemade mixtures should not be recommended because preparation errors and contamination may cause tissue injury or ineffective treatment.

Honey‐based products should be limited to sterile, standardized medical‐grade dressings used for a clinician‐selected indication. Reviews suggest antimicrobial and autolytic activity in some chronic and burn wounds, but evidence is heterogeneous and does not demonstrate broad superiority to standard care [16, 17]. Raw, farmers‐market, or food‐grade honey is nonsterile, may contain microbial spores including C. botulinum, and is not standardized; it must not be applied to an open wound.

Essential oils are sometimes promoted for antimicrobial or anti‐inflammatory effects, but most wound‐healing evidence is preclinical or derived from small, heterogeneous studies [19]. Nearly 80 essential oils have been associated with contact allergy, and composition and concentration vary across consumer products [20]. Direct application of undiluted or nonregulated essential oils to open wounds is therefore not recommended except as part of a research protocol or when using a regulated formulation prescribed for a specific indication.

The clinical evidence for aloe vera is heterogeneous, with the clearest signal in selected superficial burns rather than across all postoperative or chronic wounds [18]. If considered, clinicians should specify a standardized commercial formulation and indication. Fresh plant material and homemade preparations should not be applied to surgical, deep, infected, or complex wounds, and aloe vera should not delay formal burn or wound evaluation.

#### 3.3.3 Moisture Management and Clinician‐Delivered Adjuncts

Gauze‐based negative‐pressure wound therapy has been described in small trauma and pediatric series, but it is a clinician‐assembled therapy rather than a household substitute [22, 23]. It should be used only by trained teams in monitored clinical settings with documented pressure, wound‐bed protection, dressing counts, and follow‐up. Active bleeding, exposed or inadequately protected vessels, organs, nerves, or anastomoses, unexplored fistulae, necrotic eschar, untreated osteomyelitis, and other recognized contraindications require avoidance or specialist management [21]. Improvised NPWT should never be recommended for unsupervised home assembly.

Household plastic wrap is not equivalent to a sterile semipermeable medical film. Limited microbiologic evidence addresses temporary coverage of acute burns, not routine treatment of postoperative or chronic wounds [24]. Outside an established acute‐burn protocol, its role should be restricted to an external shower barrier over a fully sealed primary dressing when specifically approved by the treating clinician. It should not contact an open postoperative wound, remain in place for prolonged occlusion, or be used on infected or heavily exudative wounds. Clothing items should not be used as wound‐contact layers.

#### 3.3.4 Closed‐Incision Protection and Scar Management

A self‐adhering foam construct has been described as an external guard for a reconstructed nipple [25]. This narrow technical application provides mechanical protection according to the operating surgeon's protocol; it should not be generalized as a silicone‐sheet substitute or used as a primary dressing in direct contact with an open or infected wound.

Paper tape has comparative clinical support for tension offloading over selected, fully epithelialized linear incisions and may reduce hypertrophic‐scar features [26, 27]. It should not be applied to open, infected, draining, or fragile wounds. Patients with adhesive sensitivity require caution and should stop use if dermatitis, blistering, or skin stripping occurs.

Over‐the‐counter elastic wraps or athletic compression garments should not be treated as automatic substitutes for custom‐fitted medical compression. If cost limits access, a clinician should first assess the indication, arterial perfusion, sensation, skin integrity, cardiopulmonary status, fit, and target pressure, and should arrange follow‐up monitoring. Uneven or excessive pressure can cause skin, soft‐tissue, or nerve injury, particularly when perfusion or sensation is impaired [28, 29, 30].

### 3.4 Limitations and Research Gaps

This review is limited by the absence of formal screening counts and risk‐of‐bias assessment, heterogeneity across wound types, and reliance on small observational studies or expert opinion for several alternatives. Retail prices and discount programs are time‐sensitive and were not evaluated in a formal economic model. Accordingly, the review cannot establish equivalence, cost‐effectiveness, or total episode‐of‐care savings for improvised products. Household products discussed for theoretical external use have not been clinically validated; their inclusion identifies a potential option for study rather than a recommendation for routine care. Research priorities include pragmatic trials of evidence‐supported lower‐cost options, prospective safety studies of external secondary materials, standardized evaluation of clinician‐assembled NPWT in resource‐limited settings, and economic analyses that include complications, dressing frequency, labor, caregiver burden, and downstream utilization.

## 4 Conclusions

Lower‐cost wound care can improve access only when it preserves established clinical standards. Evidence currently supports a limited set of economical approaches for selected wounds, including potable tap‐water cleansing, plain petrolatum for selected clean postprocedural wounds, and paper tape for selected closed incisions. Other strategies should be confined to external secondary protection, clinician‐supervised settings, or research, and several household substances should not contact open wounds. By separating acquisition price from cost‐effectiveness and evidence‐supported care from expert opinion, clinicians can discuss affordability without normalizing unsafe or stigmatizing second‐tier care.

## Author Contributions

**Joseph D. Quick:** conceptualization, methodology, writing – original draft. **Lauren E. Powell:** conceptualization, methodology, writing – original draft. **Aneesh Syal:** writing – review and editing, project administration. **Neej N. Patel:** writing – review and editing, project administration. **Lana Kamel:** writing – review and editing. **Athena Brooks:** writing – review and editing. **Syed Ali Haider:** writing – review and editing, project administration. **Timothy A. Powell:** writing – review and editing, supervision, project administration. **Marie‐Claire Buckley:** writing – review and editing, project administration, supervision.

## Funding

The authors have nothing to report.

## Disclosure

All authors have read and approved the final version of the manuscript. Marie‐Claire Buckley, MD, the manuscript guarantor, had full access to the materials used in this narrative review and takes complete responsibility for the integrity of the work and the accuracy of the evidence synthesis.

## Conflicts of Interest

The authors declare no conflicts of interest.

## Transparency Statement

Marie‐Claire Buckley, MD, affirms that this manuscript is an honest, accurate, and transparent account of the review being reported; that no important aspects of the review have been omitted; and that any discrepancies from the review as planned have been explained.

## Data Availability

Data sharing is not applicable to this article as no datasets were generated or analyzed during the current study.
